# Greenspace and Survival Among Older Women With Breast Cancer: Regional Variations Within the U.S. SEER-Medicare-Linked Database

**DOI:** 10.1016/j.jacadv.2025.102069

**Published:** 2025-08-18

**Authors:** Jean C. Bikomeye, Emily L. McGinley, Yuhong Zhou, Sergey Tarima, Jamila L. Kwarteng, Andreas M. Beyer, Tina W.F. Yen, Aaron N. Winn, Kirsten M.M. Beyer

**Affiliations:** a Medical College of Wisconsin, Milwaukee, Wisconsin, USA; b University of Illinois at Chicago, Rockford, Illinois, USA

**Keywords:** breast cancer, cardiovascular diseases, survival, urban greenspace

## Abstract

**Background:**

Breast cancer (BC) is the most frequently diagnosed cancer among women in the United States. Cardiovascular disease (CVD) is a major noncancer cause of death among BC survivors. Although greenspace is linked to better CVD and BC-related outcomes, its effect on BC survival is unknown.

**Objectives:**

This study investigates the association between urban greenspace and survival among older BC survivors in the United States and examines regional differences.

**Methods:**

Data from the 2010 to 2017 Surveillance, Epidemiology, and End Results–Medicare BC cohort was used. Women aged 66+ with invasive BC, enrolled in Medicare (Parts A and B) for 12 months prediagnosis and with known tract-level greenspace data (N = 86,300) were included. Greenspace was measured as census tract percent tree canopy quartiles. Survival outcomes included all-cause mortality (ACM), BC-specific mortality (BCSM), and CVD-specific mortality (CVDSM), with censoring by December 31, 2018. Covariates included age, comorbidity, race/ethnicity, Medicaid eligibility, tumor stage and subtype, neighborhood social vulnerability, and population density.

**Results:**

Of 86,300 women, 22,541 (26.1%) died during the follow-up, 9,012 (40.6%) and 4,195 (18.9%) died from BC and CVD, respectively. Greater percent tree canopy was associated with lower ACM (HR: 0.90; 95% CI: 0.86-0.95) and BCSM (cause-specific HR: 0.90; 95% CI: 0.82-0.98). Regional variations were observed, with greenspace linked to lower ACM in California, New Jersey, and Michigan, and lower BCSM and CVDSM in California and New Jersey. Washington, Louisiana, and Georgia showed nonsignificant or inconsistent results.

**Conclusions:**

This study highlights the importance of investigating the relationship between greenspace and cardiooncology-related outcomes across regions, underscoring the need for more place-specific research to guide targeted interventions to improve survival outcomes.

Breast cancer (BC) is the most frequently diagnosed cancer and the leading cause of death (CoD) among women with BC.^1^^,^^2^ Among cancer-related deaths among women in the United States overall, BC is the second-leading CoD.^3^ The American Cancer Society estimates that in 2025, BC among women will account for 32% of all new cancer cases (n = 316,950) and contribute to 14% of cancer-related deaths (n = 42,170).^4^ Overburdened by multiple comorbidities, older women face a disproportionate impact of cancer-related deaths.^5^ Fortunately, advancements in early detection and treatment have had a positive impact on BC outcomes, including on survival^6^; with over 4 million BC survivors in the United States.^7^ The 5-year survival rates for women with BC have improved, increasing from 75% in the period of 1975 to 1977 to 91% in the years 2013 to 2019.^8^

However, despite these advancements, BC remains the leading CoD, particularly within the first 10 years following diagnosis. In a 15-year follow-up study of women with BC in the United States (2000-2015), using data from the Surveillance, Epidemiology, and End Results (SEER) registries representing nearly 30% of the U.S. population, BC was identified as the primary CoD within the first year (65.4%), 1 to 5 years (58.6%), and 5 to 10 years (38.2%) following BC diagnosis.^9^ Within the same study, cardiovascular disease (CVD) was the most prevalent noncancer CoD within the first year (12.4%), 1 to 5 years (13.9%), and 5 to 10 years (19.6%) following BC diagnosis,^9^ with cancer treatment–related cardiotoxicity contributing to this elevated prevalence.^10^ Beyond the 10-year mark, CVD becomes the leading CoD (24.5%).^9^ Furthermore, other SEER data have shown that among women with BC, CVD competes with BC as the leading noncancer CoD.^11^^,^^12^ BC survivors face an elevated risk of CVD mortality compared to women without BC.^13^ This risk is particularly pronounced among older women at BC diagnosis and among Black women, who face the highest risk of CVD mortality.^11^^,^^14^

Improvement in survival among BC survivors over the past 4 decades^8^ calls for nuanced approaches to maintain progress and enhance the quality of BC survivorship. Social determinants of health, including neighborhood socioenvironmental conditions in which people are born, live, learn, work, play, worship, and age, are crucial targets for intervention to enhance BC outcomes including survival.^15^ Notably, neighborhood greenspace has been associated with improved health outcomes including lower all-cause mortality (ACM)^16^^,^^17^ and improved cancer-related and CVD outcomes.^18^, ^19^, ^20^ In line with this literature, investing in urban greenspace has been proposed as an essential intervention to reduce health disparities, and improve health equity, particularly for chronic diseases, including cancer and CVD.^21^^,^^22^

Associations between greenspace and positive health outcomes have long been established via various biological pathways.^16^, ^17^, ^18^^,^^23^, ^24^, ^25^, ^26^, ^27^ Nevertheless, the impact of greenspace on survival among women with BC remains unknown. In this paper, we aim to investigate the relationship between urban neighborhood greenspace and survival from ACM, CVD-specific mortality (CVDSM), and BC-specific mortality (BCSM), among older women with BC in the United States, using data from SEER and Medicare (SEER-Medicare) linked database (2010-2017). Furthermore, we explore regional variations that may arise from varying characteristics of urban areas, including differences in city size, land use, population density, development intensity, differences in greenness levels, types of greenspace usage, and sociodemographic characteristics, using selected larger SEER registries. We hypothesize that living in a census tract with greater percent tree canopy will be associated with better survival but that associations will differ by CoD and SEER region.

## Methods

### Data sets

This cohort study, approved by the Medical College of Wisconsin Institutional Review Board, used data from women newly diagnosed with invasive BC (2010-2017) from the SEER-Medicare linked database. Patient data were linked to 2010 census tract boundaries to determine patient’s residential census tract at BC diagnosis. Tract-level measures of greenspace (2011 National Landcover Database data on tree canopy), population density, and social vulnerability (Center for Disease Control [CDC] Social Vulnerability Index [SVI] for 2010) were linked to characterize women’s residential environments.

### Inclusion criteria

**Figure 1:**
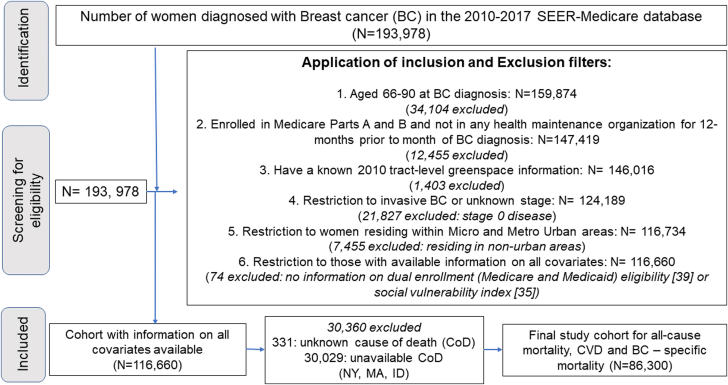
Cohort Screening Flowchart: Inclusion and Exclusion Criteria The cohort identification process resulted in a final study population of 86,300 women, selected from an initial 193,978 breast cancer diagnoses in the SEER-Medicare database (2010-2017), after applying all inclusion criteria. BC = breast cancer; CoD = cause of death; CVD = cardiovascular disease; SEER = Surveillance, Epidemiology, and End Results.

This study included women aged 66 to 90 years, diagnosed with their first invasive BC during 2010 to 2017, and alive at diagnosis. To calculate comorbidity, the cohort was restricted to women enrolled in Medicare parts A and B and not in any health maintenance organization for 12 months before BC diagnosis. Only women with known tract-level greenspace information, residing in urban areas (defined as combined statistical areas: metropolitan statistical areas and micropolitan statistical areas), with all covariates information were included (N = 86,300). Cases from registries where CoD information was unavailable or unknown, specifically Idaho, Massachusetts, and New York, were excluded from the analysis. The cohort identification process is illustrated in Figure 1.

### Variables

#### Outcome variables

Outcome variables are survival from ACM, BCSM, and CVDSM. The BC-specific CoD indicator was created using the CDC CoD coding manual and included these International Classification of Diseases (ICD)-10 codes: C50x, D05x, D486. CVD-specific death was defined using ICD codes for heart diseases (I00-I09, I11, I13, I20-I51), cerebrovascular diseases (I60-I69), atherosclerosis (I70), and other arterial diseases (I72-I78), consistent with previous studies.^28^, ^29^, ^30^ The list of all ICD codes are available in Supplemental Table 1.

#### Independent variable

The independent variable, percent tree canopy in the census tract of residence at BC diagnosis, was derived from the National Landcover Database 30-m resolution tree canopy estimates for year 2011. The percent tree canopy was defined using the state-specific percent tree canopy cover quartiles.

#### Covariates

Covariates were obtained from SEER-Medicare and are well-established factors known to impact BCSM^11^ and survival.^2^ Age at diagnosis was categorized into the following 4 groups: 66 to 70, 71 to 75, 76 to 80, and 81 to 90 years. The combined hormone receptor (estrogen or/and progesterone) and human epidermal growth factor receptor 2 (HER2) tumor receptor status was classified into the following five categories: hormone receptor positive and HER2 positive (HR+/HER2+), hormone receptor positive and HER2 negative (HR+/HER2−), hormone receptor negative and HER2 positive (HR−/HER2+), hormone receptor negative and HER2 negative (HR−/HER2−, triple negative), and unknown. The tumor stage classification followed the American Joint Committee on Cancer TNM guidelines sixth edition, stage 0 cancers were excluded.

Race/ethnicity was derived from a combination of SEER race and ethnicity variables and defined as a 4-category variable. Individuals identified as Hispanic were grouped into one category. Non-Hispanic individuals were categorized by racial category, with separate groups for non-Hispanic Black (NHB) and non-Hispanic White (NHW) women. Asian, Pacific Islander, native American, and other racial groups were aggregated into one non-Hispanic other group due to small numbers in these categories and the desire to include these individuals in the study. Comorbidity was calculated using inpatient, outpatient, and carrier Medicare claims data during the 12 months before incident BC diagnosis using the Klabunde algorithm (grouped as: none, 1, and ≥2).^31^ Dual enrollment (Medicare and Medicaid) eligibility was used as a proxy for individuals' socioeconomic status (SES) and dichotomized into eligible or not.

The tract-level population density for 2010 was included and log transformed due to a quadratic relationship between population density and percent tree canopy. The 2010 tract level SVI was used as a measure of census tract relative vulnerability, capturing potential negative effects of external health stressors by ranking tracts on 14 social risk factors.^32^ SVI was dichotomized into high vulnerability (tracts at or above the 90th percentile) or low vulnerability (tracts below the 90th percentile) according to CDC established guidelines.^32^

### Statistical analysis

Statistical analyses were performed using Stata SE (17.0, StataCorp), with significance level set at alpha = 0.05. Descriptive statistics and chi-square tests were used to summarize the study cohort characteristics based on percent tree canopy quartiles. Cox proportional hazards regression models, adjusted for state clustering to obtain national estimates and census tract clustering for state-specific estimates, were used to model time-to-death for ACM, BCSM, and CVDSM across SEER registries of California, Connecticut, Georgia, Iowa, Kentucky, Louisiana, Michigan (Detroit metropolitan area), New Jersey, New Mexico, Utah, and Washington (Seattle-Puget Sound metropolitan area). In addition, state-specific analyses were conducted for selected larger SEER registries, including California, Detroit, Georgia, Louisiana, New Jersey, and Washington. Log-rank tests were used to compare 1- and 5-year survival between different tree canopy quartiles grades.

Kaplan-Meier and Aalen-Johansen methods were used to estimate survival probability and probability of BCSM and CVDSM at different postdiagnosis times, respectively. All Cox regression models adhered to the proportionality of hazards assumption, with covariate stratification applied as needed. Survival time was calculated using the date of death from the Medicare enrollment file for deceased patients, censoring all individuals alive on December 31, 2018 for BCSM and CVDSM, based on vital status from Medicare and CoD information from SEER. The censoring date of 12/31/2018 was necessary to ensure cohort compatibility across all outcomes. Data for ACM were truncated at year 6 (72 months) from the BC diagnosis date to maintain the proportionality assumption for fitting Cox models.

## Results

### Sample demographics and tumor characteristics

Table 1 provides an overview of sample demographics and tumor characteristics by percent tree canopy quartiles (N = 86,300). The mean percent tree canopy quartiles are as follows: 6.9% ±10.4%, 14.8% ±14.9%, 23.0% ±18.6%, and 39.6% ±21.7 v, respectively, from the lowest (first) to the highest (fourth) quartile. Majority of women identified as NHW (82.0%), with a higher proportion residing in tracts within the highest quartile of percent tree canopy (90.3%) and 72.4% residing in the lowest quartile. In contrast, NHB women comprised 7.8% of the cohort, with only 3.7% residing in tracts within the highest quartile and 13.0% in tracts within the lowest quartile. The mean population density is lowest in tracts with the highest percent tree canopy (1,411.2 vs 6,724.8 in tracts within the lowest percent tree canopy quartile). Medicare and Medicaid dual enrollment eligible women comprised 13.5% of the cohort, with only 8.9% residing in tracts within the highest quartile and 19.7% in the lowest quartile.

**Table 1:** Demographic Characteristics of the 2010-2017 SEER-Medicare BC Cohort: Censoring Date in December 31, 2018

	Total (N = 86,300)	First Quartile (n = 21,593, 25.0%)	Second Quartile (n = 21,586, 25.0%)	Third Quartile (n = 21,604, 25.0%)	Fourth Quartile (n = 21,517, 25.0%)	*P* Value
Percent tree canopy cover (mean ± SD)	21.0 (±20.8)	6.9 (±10.4)	14.8 (±14.9)	23.0 (±18.6)	39.6 (±21.7)	<0.001
Survival probability for all-cause mortality						<0.001
1 y survival (95% CI)		0.93 (0.92-0.93)	0.94 (0.93-0.94)	0.94 (0.93-0.94)	0.94 (0.94-0.94)	
5 y survival (95% CI)		0.69 (0.68-0.70)	0.70 (0.69-0.71)	0.72 (0.71-0.73)	0.74 (0.73-0.74)	
Age (mean ± SD)	75.2 (±6.5)	75.4 (±6.6)	75.3 (±6.6)	75.3 (±6.5)	74.9 (±6.5)	<0.001
Age at BC diagnosis, y, n (%)						<0.001
66-70	25,868 (30.0)	6,226 (28.8)	6,442 (29.8)	6,442 (29.8)	6,758 (31.4)	
71-75	22,567 (26.1)	5,587 (25.9)	5,635 (26.1)	5,577 (25.8)	5,768 (26.8)	
76-80	17,554 (20.4)	4,465 (20.7)	4,338 (20.1)	4,521 (20.9)	4,230 (19.7)	
81-90	20,311 (23.5)	5,315 (24.6)	5,171 (23.9)	5,064 (23.5)	4,761 (22.1)	
Race and ethnicity, n (%)						<0.001
NHW	70,768 (82.0)	15,629 (72.4)	17,273 (80.0)	18,426 (85.3)	19,440 (90.3)	
NHB	6,745 (7.8)	2,808 (13.0)	1,844 (8.5)	1,290 (6.0)	803 (3.7)	
NHO	3,606 (4.2)	1,134 (5.3)	1,040 (4.8)	845 (3.9)	587 (2.7)	
Hispanic	5,181 (6.0)	2,022 (9.4)	1,429 (6.6)	1,043 (4.8)	687 (3.2)	
Comorbidity, n (%)						<0.001
None	42,688 (49.5)	9,802 (45.4)	10,331 (47.9)	10,986 (50.8)	11,569 (53.8)	
1	22,696 (26.3)	5,755 (26.7)	5,621 (26.0)	5,719 (26.5)	5,601 (26.0)	
2+	20,916 (24.2)	6,036 (27.9)	5,634 (26.1)	4,899 (22.7)	4,347 (20.2)	
Tumor stage (TNM stage) at diagnosis (n (%)						<0.001
Stage I	46,011 (53.3)	11,000 (50.9)	11,461 (53.1)	11,625 (53.8)	11,925 (55.4)	
Stage II	24,439 (28.3)	6,328 (29.3)	6,052 (28.0)	6,181 (28.6)	5,878 (27.3)	
Stage III	7,223 (8.4)	1,955 (9.0)	1,831 (8.5)	1,775 (8.2)	1,662 (7.7)	
Stage IV	5,262 (6.1)	1,367 (6.3)	1,403 (6.5)	1,202 (5.6)	1,290 (6.0)	
Unknown stage	3,365 (3.9)	943 (4.4)	839 (3.9)	821 (3.9)	762 (3.5)	
BC diagnosis year n (%)						<0.001
2010	10,598 (12.3)	2,744 (12.7)	2,766 (12.8)	2,558 (11.8)	2,530 (11.7)	
2011	10,649 (12.3)	2,793 (12.9)	2,634 (12.2)	2,635 (12.0)	2,587 (12.0)	
2012	10,740 (12.4)	2,759 (12.8)	2,671 (12.4)	2,696 (12.5)	2,614 (12.1)	
2013	10,771 (12.5)	2,660 (12.3)	2,719 (12.6)	2,684 (12.4)	2,708 (12.6)	
2014	10,789 (12.5)	2,649 (12.3)	2,729 (12.6)	2,726 (12.6)	2,685 (12.5)	
2015	10,916 (12.6)	2,653 (12.3)	2,647 (12.3)	2,779 (12.8)	2,837 (13.2)	
2016	11,024 (12.8)	2,746 (12.7)	2,726 (12.6)	2,798 (13.9)	2,754 (12.8)	
2017	10,813 (12.5)	2,589 (12.0)	2,694 (12.5)	2,728 (12.6)	2,802 (13.0)	
Hormone receptor and tumor-receptor-status, n (%)						<0.001
HR+/HER2−	62,885 (72.9)	15,362 (71.1)	15,521 (71.9)	15,976 (74.0)	16,026 (74.5)	
HR−/HER2− (triple negative)	7,250 (8.4)	1,959 (9.1)	1,836 (8.5)	1,757 (8.1)	1,698 (7.9)	
HR+/HER2+	6,368 (7.4)	1,571 (7.3)	1,628 (7.6)	1,629 (7.5)	1,540 (7.2)	
HR−/HER2+	2,495 (2.9)	656 (3.0)	655 (3.0)	585 (2.7)	599 (2.8)	
Unknown	7,302 (8.5)	2,045 (9.5)	1,946 (9.0)	1,657 (7.7)	1,654 (7.7)	
Dual eligibility to Medicare and Medicaid, n (%)						<0.001
Eligible (Poorer)	11,679 (13.5)	4,249 (19.7)	3,138 (14.5)	2,375 (11.0)	1,917 (8.9)	
Not eligible	74,621 (86.5)	17,344 (80.3)	18,448 (85.5)	19,229 (89.0)	19,600 (91.1)	
SVI, n (%)						<0.001
Low vulnerability	80,368 (93.1)	18,404 (85.2)	19,910 (92.3)	20,853 (96.5)	21,201 (98.5)	
High vulnerability	5,932 (6.9)	3,189 (14.8)	1,676 (7.8)	751 (3.5)	316 (1.5)	
Population density (mean ± SD)	3,932.8 (±5,892.5)	6,724.8 (±9,255.0)	4,508.5 (±4,981.6)	3,078.5 (±3,096.9)	1,411.2 (±1880.5)	<0.001
Vital status, n (%): Follow-up ending at year 6						<0.001
Alive	63,759 (73.9)	15,448 (71.6)	15,804 (73.2)	16,116 (74.6)	16,391 (76.2)	
Deceased	22,541 (26.1)	6,145 (28.5)	5,782 (26.8)	5,488 (26.4)	5,126 (23.8)	
Cause of death^a^, n (%)						0.305
Breast cancer	9,012 (40.6)	2,538 (42.6)	2,289 (39.9)	2,169 (40.4)	2,026 (40.4)	
Other causes	13,178 (59.4)	3,552 (58.4)	3,444 (60.1)	3,193 (59.6)	2,989 (59.6)	
Cause of death^a^, n (%)						0.024
Cardiovascular disease	4,194 (18.9)	1,095 (18.0)	1,149 (20.1)	1,031 (19.2)	919 (18.3)	
Other causes	17,996 (81.1)	4,985 (82.0)	4,584 (79.9)	4,331 (80.8)	4,096 (81.7)	

BC = breast cancer; CoD = cause of death; CVD = cardiovascular disease; HER2 = human epidermal growth factor receptor 2; NHB = non-Hispanic Black; NHO = non-Hispanic other; NHW = non-Hispanic White; NLCD = National land cover database; SVI = Social Vulnerability Index.

a Among the n = 22,190 with CoD information.

A predominant number of women were diagnosed with HR+/HER2-tumors (72.9%), were stage I (53.3%), and had no comorbidity (49.5%). Meanwhile, 26.3% had 1 comorbid condition, and 24.2% had 2+ comorbid conditions. With a mean age of 75.2 ±6.5 years, 30.0% of women were in the 66 to 70 age group, 26.1% in the 71 to 75 age group, 20.4% in the 76 to 80 age group, and 23.5% in the 81 to 90 age group. The year of BC diagnosis for women was evenly distributed, ranging from 12.3% in 2010 to 12.8% in 2016. Most women lived in areas with low social vulnerability (93.1%), a larger proportion of whom residing primarily in neighborhoods with high tree canopy coverage (98.5% in the fourth quartile). Conversely, 6.9% lived in areas with high social vulnerability, with a significant portion of these (14.8%) in the lowest tree canopy quartile, and only 1.5% in the highest quartile.

At the study period’s end, with a median survival time of 32 months, 26.1% of women were deceased (n = 22,541). Among those with a known CoD (n = 22,190), a higher proportion died from BC (n = 9,012; 40.6%), whereas 4,194 (18.9%) died from CVD. One-year survival did not differ across percent tree canopy quartiles; however, 5-year survival significantly varied across quartiles, with survival probabilities and 95% CI of 0.69 (0.68-0.70), 0.70 (0.69-0.71), 0.72 (0.71-0.73), and 0.74 (0.73-0.74) from the lowest to the highest quartiles, respectively. Bivariate analysis revealed no significant difference in BCSM among quartiles. However, for CVDSM, the second quartile showed a slightly higher proportion of deaths from CVD (20.1% vs 18.9%).

### Model findings: full cohort and sub-group analyses by seer region

Table 2 provides a summary of Cox Proportional Hazards Models, examining the adjusted effect of percent tree canopy quartiles on time to death from any cause, BCSM, and CVDSM. Associations between greenspace and ACM and BCSM were statistically significant. Adjusted HR (AHR) and cause-specific HR (CSHR, 95% CI) indicated a lower risk of ACM and BCSM for women residing in the highest percent tree canopy quartile compared to those in the lowest quartile: 0.90 (0.86-0.95) and 0.90 (0.82-0.98), respectively. Overall results for CVDSM were not statistically significant.

**Table 2:** Cox Models Exploring Associations Between Greenspace and Time to Death From ACM, BCSM, and CVDSM

	All-Cause Mortality Stratified and Adjusted	BC Specific Mortality Stratified and Adjusted	CVD Specific Mortality Stratified and Adjusted
	HR (95% CI)	CSHR (95% CI)	CSHR (95% CI)
Greenspace quartiles (mean ± SD)			
Q1: 6.9 (±10.4); lowest)	REF	REF	REF
Q2: 14.8 (±14.9)	**0.97 (0.94-1.00)**^a^	0.91 (0.86-1.96)	1.09 (0.96-1.16)
Q3: 23.0 (±18.6)	**0.97 (0.94-0.99)**^a^	0.95 (0.90-1.01)	1.03 (0.96-1.10)
Q4: 39.6 (±21.7); highest)	**0.90 (0.86-0.95)**^a^	**0.90 (0.82-0.98)**^a^	0.94 (0.82-1.09)
Dual eligibility to Medicare and Medicaid			
Not eligible	REF	REF	REF
Eligible (poorer)	1.42 (1.34-1.51)^a^	1.35 (1.26-1.44)^a^	1.52 (1.34-1.73)^a^
Neighborhood SVI			
Low vulnerability	REF	REF	REF
High vulnerability	1.07 (1.01-1.13)^a^	1.15 (1.08-1.22)^a^	0.99 (0.88-1.11)
Race and ethnicity			
NHW	REF	REF	REF
NHB	1.00 (0.96-1.06)	1.11 (1.07-1.16)^a^	1.02 (0.93-1.13)
NHO	0.57 (0.52-0.63)^a^	0.64 (0.58-0.71)^a^	0.49 (0.43-0.56)^a^
Hispanic	0.74 (0.69-0.80)^a^	0.83 (0.76-0.92)^a^	0.65 (0.53-0.78)^a^
Comorbidity			
None	REF	REF	REF
One	1.36 (1.31-1.42)^a^	1.07 (1.02-1.13)^a^	1.80 (1.66-1.97)^a^
Two or more	2.25 (2.15-2.36)^a^	1.37 (1.30-1.44)^a^	3.63 (3.38-3.89)^a^

All models are adjusted for race/ethnicity, SVI, dual enrollment (Medicare and Medicaid) eligibility, log transformed tract-level population density, comorbidity, and stratified by diagnosis year, stage, age, and tumor subtype. All models are adjusted for random error by state clusters. **Bold** values indicate statistically significant.

ACM = all-cause mortality; BCSM = breast cancer–specific mortality; CSHR = cause specific–HR; CVD = cardiovascular disease; CVDSM = cardiovascular disease–specific mortality; other abbreviations as in Table 1.

a Denotes statistical significance (*P* ≤ 0.05).

Significant effects were observed for covariates: state of residence, dual enrollment eligibility, SVI, race/ethnicity, and comorbidity. In addition, a significant interaction was found between tree canopy and state of residence (Supplemental Table 2), whereas no interactions were observed for race/ethnicity, dual enrollment eligibility, or SVI. Subsequently, to examine state-specific greenspace relationships, subgroup analyses were performed by state of residence using larger SEER state registries from various U.S. regions, including California, Washington, New Jersey, Georgia, Louisiana, and Michigan. Significant variations across SEER regions were observed (Table 3).

**Table 3:** Cox Models Exploring Associations Between Greenspace and Time to Death Across Larger SEER Regions

SEER Registries (Region)	Percent Tree Canopy Quartiles	All-Cause Mortality Stratified and Adjusted	BC Specific Mortality Stratified and Adjusted	CVD Specific Mortality Stratified and Adjusted
		HR (95% CI)	CSHR (95% CI)	CSHR (95% CI)
California (West Coast) (N = 29,071)	Q1 (lowest)	REF	REF	REF
	Q2	0.96 (0.90-1.01)	**0.89 (0.80-0.98)**^a^	1.07 (0.95-1.21)
	Q3	0.98 (0.91-1.05)	0.95 (0.89-1.02)	0.97 (0.88-1.08)
	Q4 (highest)	**0.92 (0.85-0.99)**^a^	**0.86 (0.77-0.97)**^a^	**0.80 (0.69-0.94)**^a^
Washington (West Coast) (N = 5,513)	Q1 (lowest)	REF	REF	REF
	Q2	1.00 (0.94-1.06)	1.23 (0.99-1.53)	1.05 (0.98-1.13)
	Q3	0.98 (0.91-1.07)	1.02 (0.82-1.25)	1.03 (0.88-1.22)
	Q4 (highest)	1.06 (0.89-1.27)	1.40 (0.86-2.23)	0.87 (0.52-1.45)
New Jersey (East Coast) (N = 14,594)	Q1 (lowest)	REF	REF	REF
	Q2	0.99 (0.96-1.02)	**0.89 (0.82-0.97)**^a^	1.15 (0.99-1.33)
	Q3	**0.96 (0.92-0.99)**^a^	0.95 (0.84-1.07)	0.99 (0.93-1.06)
	Q4 (highest)	**0.86 (0.82-0.90)**^a^	**0.82 (0. 74-0.90)**^a^	0.91 (0.70-1.11)
Georgia (South) (N = 9,590)	Q1 (lowest)	REF	REF	REF
	Q2	1.02 (0.95-1.10)	0.95 (0.81-1.11)	*1.38 (1.14-1.66)*^a^
	Q3	1.05 (0.96-1.15)	1.06 (0.88-1.27)	*1.34 (1.11-1.62)*^a^
	Q4 (highest)	*1.14 (1.03-1.26)*^a^	1.04 (0.86-1.25)	*1.49 (1.18-1.87)*^a^
Louisiana (South) (N = 5,202)	Q1 (lowest)	REF	REF	REF
	Q2	0.99 (0.89-1.09)	0.84 (0.62-1.15)	0.87 (0.64-1.19)
	Q3	1.06 (0.93-1.22)	1.07 (0.80-1.44)	0.86 (0.62-1.19)
	Q4 (highest)	1.05 (0.87-1.28)	1.25 (0.92-1.68)	0.80 (0.53-1.20)
Michigan (Midwest) (N = 4,914)	Q1 (lowest)	REF	REF	REF
	Q2	0.98 (0.83-1.15)	1.18 (0.90-1.54)	0.93 (0.67-1.30)
	Q3	0.88 (0.73-1.05)	0.99 (0.73-1.34)	1.07 (0.75-1.55)
	Q4 (highest)	**0.78 (0.63-0.96)**^a^	0.91 (0.63-1.30)	0.81 (0.53-1.26)

All models are adjusted for race, SVI, dual enrollment (Medicare and Medicaid) eligibility, tract-level population density, comorbidity, and stratified by diagnosis year, stage, age, and tumor subtype. All models are adjusted for random error by census tracts clusters, except Michigan that has no tract clusters.

Abbreviations as in Table 1, Table 2.

a Statistical significance (*P* ≤ 0.05), bold in hypothetical direction, italic in the counterintuitive direction.

In California, an association between tree canopy and ACM, BCSM, and CVDSM was observed. The AHR (95% CI) for ACM was 0.92 (0.85-0.99) for women residing in the highest percent tree canopy quartile compared to women in the lowest. Adjusted CSHR (95% CI) for BCSM and CVDSM were 0.86 (0.77-0.97) and 0.80 (0.69-0.94), respectively, for women in the highest percent tree canopy quartile compared to those in the lowest.

In New Jersey and Michigan, an association between greenspace and ACM was observed, with AHR (95% CI) of 0.86 (0.82-0.90) and 0.78 (0.63-0.96), respectively. Furthermore, in New Jersey, an association was observed for BCSM, with CSHR (95% CI) of 0.82 (0.74-0.90). However, although the CSHRs for these states suggested a decrease in CVDSM, these results were not statistically significant. Similarly, BCSM results in Michigan were not significant. In Washington and Louisiana, there were no statistically significant findings.

Results were different in Georgia, with more tree canopy associated with an increased risk for ACM, with AHR (95% CI) of 1.14 (1.03-1.26), and CVDSM, with CSHR (95% CI) of 1.49 (1.18-1.87), indicating a higher risk for women residing within the highest quartile of greenspace compared to those in the lowest. Results were not significant for BCSM.

### Kaplan-Meier and Aalen-Johansen estimates

**Figure 2:**
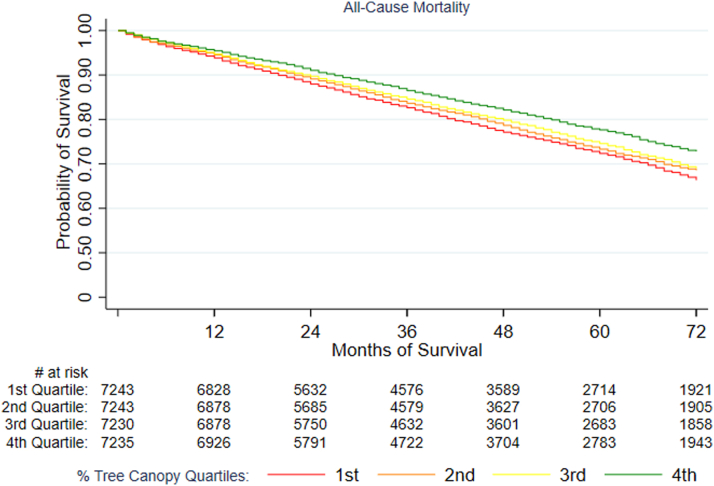
Kaplan-Meier Estimate of the Survival Function for ACM: SEER-Medicare BC Cohort (2010-2017) Women residing in the highest percent tree canopy quartile (green line) exhibited better overall survival than those in the lowest percent tree canopy quartiles (red line). ACM = all-cause mortality; other abbreviations as in Figure 1.

**Figure 3:**
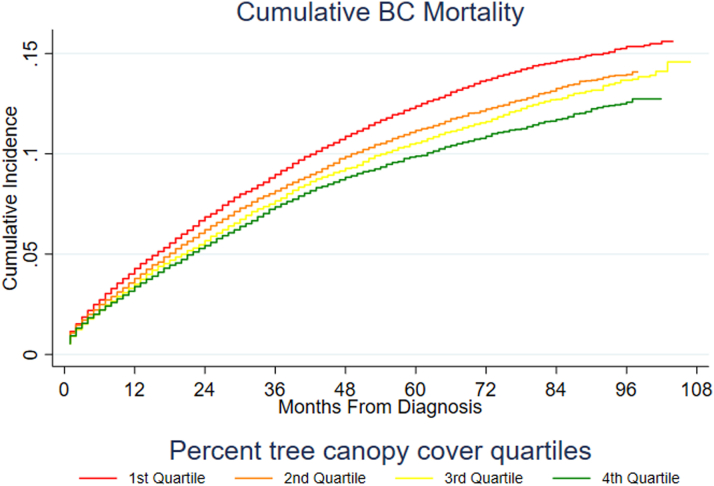
Aalen-Johansen Estimate of Cumulative Incidence Function for BCSM: SEER-Medicare BC Cohort (2010-2017) The Aalen-Johansen estimate of cumulative incidence function for BCSM suggests that women residing in the highest percent tree canopy quartile (green line) had lower cumulative BC mortality than those in the lowest percent tree canopy quartile (red line). BCSM = breast cancer–specific mortality; other abbreviations as in Figure 1.

**Figure 4:**
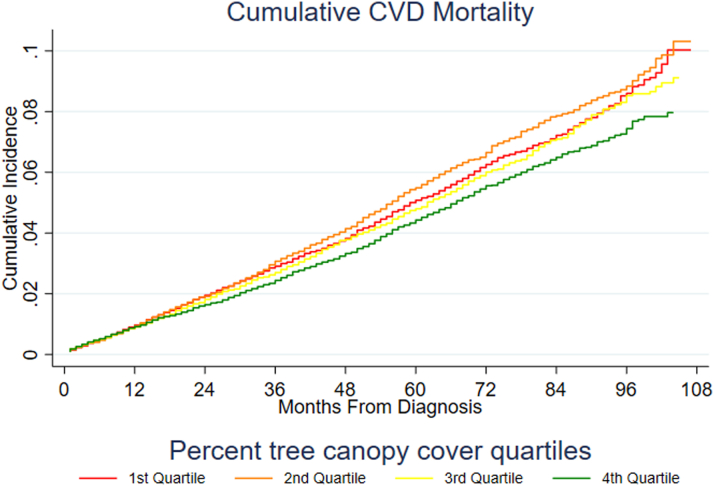
Aalen-Johansen Estimate of Cumulative Incidence Function for CVDSM: SEER-Medicare BC Cohort (2010-2017) The Aalen-Johansen estimate of cumulative incidence function for CVDSM suggests that women residing in the highest percent tree canopy quartile (green line) had lower cumulative CVD mortality than those in lower percent tree canopy quartiles. CVDSM = cardiovascular disease–specific mortality; other abbreviation as in Figure 1.

Unadjusted Kaplan-Meier estimates of the survival function for ACM and Aalen-Johansen estimates of cumulative incidence for BCSM and CVDSM for the overall cohort (n = 86,300) are shown, respectively, in Figure 2, Figure 3, Figure 4 and Central Illustration, revealing differences by tree canopy quartiles. Women in the highest percent tree canopy quartile (green line) exhibited better survival than those in the lowest percent tree canopy quartiles (red line), as illustrated in Figure 2. Similarly, women in the highest percent tree canopy quartile (green line) had lower cumulative BC mortality (Figure 3) and lower cumulative CVD mortality (Figure 4 and Central Illustration) than those in lower percent tree canopy quartiles.

**Central Illustration:**
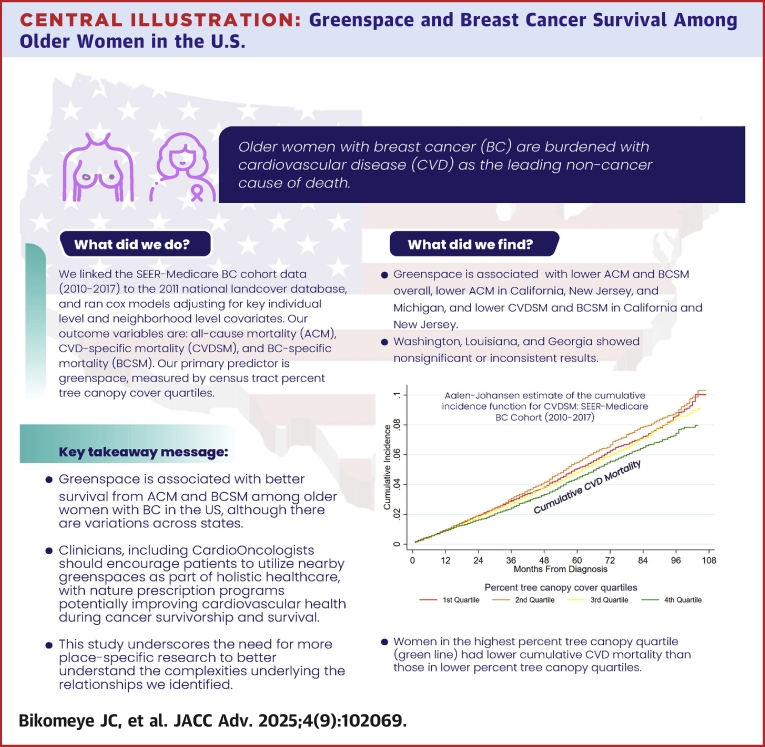
Greenspace and Breast Cancer Survival Among Older Women in the U.S. The graphical abstract illustrates the association between greenspace and survival among older women with breast cancer. It highlights that greenspace is associated with lower overall ACM and BCSM, as well as lower ACM in California, New Jersey, and Michigan, and lower BCSM and CVDSM in California and New Jersey. However, nonsignificant or inconsistent results were observed in Washington, Louisiana, and Georgia. The Aalen-Johansen estimate of cumulative incidence function for CVDSM suggests that women residing in the highest percent tree canopy quartile (green line) had lower cumulative CVD mortality than those in lower percent tree canopy quartiles. SEER = Surveillance, Epidemiology, and End Results.

## Discussion

This study examined the relationship between percent tree canopy cover quartiles and survival among older women with invasive BC in the United States (2010-2017). Associations between quartiles of tree canopy cover and survival outcomes (ACM, BCSM, and CVDSM) were examined, while also exploring variations across SEER regions. The findings revealed that higher tree canopy cover is associated with lower ACM and BCSM, but not CVDSM. Women residing in areas with the highest tree canopy cover exhibited a significant survival advantage, with 10% lower HR for ACM and 10% lower CSHR for BCSM, compared to those in areas with the lowest canopy cover. NHW women predominated the study cohort (82%), with a majority residing in areas with a high tree canopy cover (90.3%), highlighting racial/ethnic disparities in residential urban greenspace access. Conversely, NHB women (7.8%) were underrepresented in areas with a high canopy cover (3.7%), also reinforcing unequal distribution of greenspace, its access and its potential contribution to longstanding racial/ethnic health disparities in BC and CVD outcomes with NHB individuals being mostly impacted, as noted in previous studies.^22^^,^^33^

Furthermore, our study revealed variations in the associations between tree canopy cover and survival among women with BC across different SEER regions. In California, residing in areas within the highest quartile of percent tree canopy is a statistically significant predictor of better survival outcomes; women in these areas exhibit an 8% lower HR for ACM, a 14% lower CSHR for BCSM, and a 20% lower CSHR for CVDSM compared to those in the lowest quartile of percent tree canopy. Similar trends were observed in New Jersey and Michigan. In New Jersey, women in areas within the highest quartile of tree cover had a 14% lower HR for ACM, whereas in Michigan, the reduction was 22% compared to women in areas within the lowest quartile. In addition, women residing in areas within the highest quartiles in New Jersey had an 18% lower CSHR for BCSM, compared to women in the lowest quartile. These findings underscore the health benefits associated with urban greenspace, as observed in previous studies.^18^^,^^21^^,^^27^ However, no significant differences were observed for BCSM in Michigan and for CVDSM in New Jersey and Michigan. Overall, no significant associations were detected for the states of Washington or Louisiana.

Results were different in Georgia, where an unexpected relationship between greenspace and survival emerged. Counter-intuitively, a higher tree canopy was associated with poorer survival outcomes. Women living in areas within the highest quartile of percent tree canopy exhibited a 14% higher HR for ACM and a 49% higher CSHR for CVDSM compared to those in areas within the lowest quartile. One potential explanation for these unexpected results may be attributed to the lack of Medicaid expansion in the state, which limited individuals' access to additional Medicaid insurance and consequently to health care services, thus contributing to poorer survival.^34^^,^^35^ Although we controlled for dual enrollment eligibility, its effect is less impactful due to the absence of Medicaid expansion. In addition, the notably higher average percent tree canopy cover in Georgia, with the lowest quartile mean at 30.5%, compared to the overall lowest quartile at 5.9%, may have influenced these findings. No significant results were found for BCSM.

Various factors may contribute to regional variations in the statistical significance of our findings, leading to diverse research and policy implications. These factors include variations in the level of urbanization and urbanicity across different urban areas studied,^36^ differences in the distribution of tree canopy cover and neighborhood income,^37^ the quality and individual’s perceived safety of canopied spaces, the accessibility of tree canopy areas for neighborhood residents,^38^ the proximity of individual homes to greenspaces, and the actual use of these greenspaces.^39^

Recent research has suggested that individuals residing in highly urban areas may get more health benefits from trees than those in less urbanized areas.^40^ Moreover, there is evidence suggesting that shrubs may differ from trees in their ability to remove air pollution and subsequently offer distinct protective effects.^41^ However, this study did not investigate this level of granularity, highlighting an opportunity for future research. Such investigations could provide valuable insights into the specific mechanisms by which different types of vegetation influence health outcomes. By understanding these nuances, we can develop more targeted interventions aimed at maximizing the health benefits associated with greenspace exposure.

In addition, individual factors, such as SES and travel time to greenspaces, play a role in greenspace utilization. Individuals who visit public greenspaces often prefer visiting spaces within a shorter travel time from their residence.^42^ A recent review highlighted that individuals with lower SES tend to favor greenspaces closer to home due to limitations in private recreational resources, deriving more health benefits from these spaces.^22^ However, greenspaces in lower SES areas may have lower quality, be less maintained, and less safe compared to those in more privileged areas,^43^ which might potentially explain the findings observed in Georgia. Unfortunately, due to the inherent nature of the SEER-Medicare linked data set, we could not examine nuances related to greenspace-specific characteristic factors impacting its use, including safety, quality, and maintenance,^43^^,^^44^ which have all been shown to impact health outcomes.^45^ Nevertheless, our findings provide a foundational basis for future research to delve into place-specific factors that could elucidate the differences observed in this study.

Furthermore, our data set lacked more individual-level SES variables beyond dual enrollment eligibility, preventing a comprehensive assessment of SES confounding effect. Due to data set's limitations, we were unable to assess individual-level perceptions of neighborhood greenspace–specific factors that are known to impact both the willingness to use and frequency of actual use of greenspaces,^42^ such as greenspace safety and cleanliness,^21^ and proximity to greenspace or accessibility.^42^^,^^46^^,^^47^ Furthermore, greenspace can modify the relationship among anthropogenic climate change and related emissions, air pollution, urban heat island effects, and health,^21^^,^^48^^,^^49^ suggesting an important area for future investigation in relation to our findings.

The gaps discussed present avenues for future research, including qualitative and quantitative assessments of greenspace-specific factors, and individual-level perceptions of those spaces. Such research will help us better understand the role of neighborhood-level factors and individual SES in influencing the impact of greenspace on survival, which might be key driving factors influencing mixed findings reported in this paper. Further investigation is warranted to elucidate the complex interplay between regional factors and individual-specific factors that contribute to greenspace utilization, offering more insights into their ultimate impact on survival outcomes. Our findings underscore the potential of including greenspace in equity-focused interventions in efforts to address disparities in access to and utilization of greenspace and their implications for health outcomes.

### Study Limitations

Although this study used robust statistical methodologies, some limitations should be acknowledged. Firstly, although efforts were made to control for relevant covariates, the possibility of unmeasured confounding factors remains. In particular, the SEER-Medicare database lacks direct measures of individual level SES, such as income, education or employment, which would be useful to examine. To minimize the extent of this problem, we controlled for dual enrollment (Medicare and Medicaid) eligibility as a proxy for individual-level SES, and neighborhood social vulnerability. Secondly, SEER data, including patient demographics, CoD, and tumor characteristics, may have inaccuracies due to algorithms used by cancer registries, potentially leading to misclassifications, which could impact the findings—an inherent limitation of using large epidemiologic data sets. Thirdly, the study examined older women with Medicare insurance, thus excluding women with alternative insurance types and the uninsured. Fourthly, SEER-Medicare provides geographical granularity only up to census tract level, which limits the precision of greenspace exposure. In future studies with more granular geographic data, it would be valuable to use different methods of measuring greenspace and varying distances from the home. Lastly, the study’s focus on women in urban areas further restricts the generalizability of findings beyond urban settings.

Despite these limitations, this study contributes important insights into the relationship between greenspace and survival from ACM, BCSM, and CVDSM. Future investigations should incorporate more diverse samples, including younger women and those with varying insurance statuses. A deeper understanding of pathways through which greenspace influences survival outcomes will contribute to a more comprehensive understanding, helping to inform targeted interventions in the built environment that can enhance BC outcomes and survival.

### Implications for future research and policy

This study’s findings have important implications for future research and policy. Future research should investigate factors contributing to racial/ethnic disparities in greenspace exposure, as well as regional variations in associations with survival outcomes. This includes examining the quality, safety, accessibility, perceptions, and utilization of greenspaces. Such research efforts can inform evidence-based strategies to promote health equity, enhance outcomes among BC survivors, and advance intergenerational health equity. Policymakers can leverage our evidence and reduce disparities in greenspace access by investing in urban greenspaces in states with better survival outcomes linked to greenspace. This entails allocating additional resources to improve the quality, quantity, and accessibility of greenspaces, with the goal of increasing utilization, improving health outcomes, and reducing long-term health care costs linked to cancer and CV care.

## Conclusion and key takeaways

We investigated the associations between greenspace and survival among older women with BC in the United States, using SEER-Medicare data (2010-2017). Our study revealed associations between greenspace and ACM, BCSM overall, with variations observed across SEER regions. These findings underscore the need for further research to understand why greenspace is associated with better outcomes in certain areas (eg, California, New Jersey, and Michigan) while being associated with higher risks in others (eg, Georgia). Moreover, our results underscore the importance of tailored interventions and strategic investments in greenspace to enhance survivorship quality among BC survivors and the importance of considering local context in policymaking.

It is crucial to address racial/ethnic disparities in greenspace access and use and fully understand greenspace-specific factors that might have contributed to differences observed in our results. Further research is needed to uncover underlying factors and pathways, informing strategies for improving outcomes, and reducing cardiooncology-related health disparities. Furthermore, there is a need for more place-focused studies and more granular measures of greenspace as well as additional measures of SES. Nevertheless, the study highlights that urban greenspace may improve survival outcomes for older BC survivors.

Perspectives **COMPETENCY IN MEDICAL KNOWLEDGE:** Clinicians and the entire care team should encourage their patients to take advantage of greenspaces near their homes as part of holistic health care, which considers the socioenvironmental context in which individuals live. Nature prescription programs and the promotion of greenspace use could serve as valuable clinical interventions to enhance survivorship quality and improve survival.

**TRANSLATIONAL OUTLOOK:** Further research is needed to evaluate the barriers and facilitators to the uptake of these interventions, not only from the perspective of the care team but also for the individuals receiving the prescriptions and the contexts in which these interventions occur, from an implementation science perspective.

## Funding support and author disclosures

The ideas and opinions expressed herein are those of the author(s) and endorsement by the State of California Department of Public Health, the National Cancer Institute, and the Centers for Disease Control and Prevention or their Contractors and Subcontractors is not intended nor should be inferred. The work is supported by the 10.13039/100000002National Institutes of Health (NIH) grant: R01CA214805 (Dr K.M.M. Beyer), 10.13039/100000968American Heart Association Scientific focused research network on disparities in Cardio-oncology grants (Dr K.M.M. Beyer grant ID # 863108; and Dr A.M. Beyer: grant ID #: 863107), the American Heart Association Research supplement to promote diversity in Science (Dr Bikomeye; grant ID # 960133), the 10.13039/100017342Medical College of Wisconsin Cancer Center grant (Dr K.M.M. Beyer), and 10.13039/100006108National Center for Advancing Translational Sciences, NIH, Award Number UL1 TR001436 (Dr Kwarteng). All other authors have reported that they have no relationships relevant to the contents of this paper to disclose.
