# Supplementary material for: Greenspace and Survival Among Older Women With Breast Cancer: Regional Variations Within the U.S. SEER-Medicare-Linked Database
Source: JACC Adv. 2025 Aug 18;4(9):102069. doi: 10.1016/j.jacadv.2025.102069 (PMC12396096; doi:10.1016/j.jacadv.2025.102069)
Supplement: Supplementary Material [file mmc1.docx]

**Supplemental Table 1:** Variables definitions

| **Variables** | **ICD codes** |
| --- | --- |
| Breast cancer diagnosis | C50 |
| Breast cancer specific mortality | C50, C500-C509, D05, D050-D059, D24, and D486. |
| Cardiovascular diseases specific mortality   1. Heart diseases 2. Cerebrovascular diseases 3. Atherosclerosis 4. Other arterial diseases | I00–I09, I11, I13, I20–I51  I60–I69  I70  I72–I78 |

**Supplemental Table 2**: Cox Models exploring associations between greenspace interaction with state and time to death

| Variables | All-cause mortality  Stratified and adjusted | BC Specific mortality  stratified and adjusted | CVD Specific mortality  stratified and adjusted |
| --- | --- | --- | --- |
|  | Hazard ratio (95% CI) | CSHR (95% CI) | CSHR (95% CI) |
| *Greenspace quartiles(mean ± SD*)  Q1: 6.9 (±10.4); lowest)  Q2: 14.8 (±14.9)  Q3: 23.0 (±18.6)  Q4: 39.6 (±21.7); highest) | REF  0.95 (0.90 to 1.00)  0.98 (0.92 to 1.04)  0.94 (0.87 to 1.01) | REF  0.89 (0.80 to 1.00)  0.94 (0.87 to 1.01)  **0.87 (0.77 to 0.99)**** | REF  1.09 (0.96 to 1.22)  1.00 (0.90 to 1.11)  **0.84 (0.71 to 0.99)**** |
| *State of residence (n, %)*  California (29,071, 33.7%)  Connecticut (5,113, 5.9%)  Georgia (9,590, 11.1%)  Iowa (3,641, 4.2%)  Kentucky (4,901, 5.7%)  Louisiana (5,202, 6.0%)  Michigan (4,914, 5.7%)  New Jersey (14,594, 16.9%)  New Mexico (1,907, 2.2%)  Utah (1,854, 2.2%)  Washington (5,513, 6.4%) | REF  1.03 (0.90 to 1.19)  1.18 (1.08 to 1.28)**  1.27 (1.10 to 1.48)**  1.42 (1.30 to 1.55)**  1.12 (1.03 to 1.23)**  1.32 (1.24 to 1.40)**  1.01 (0.94 to 1.10)  1.19 (1.08 to 1.31)**  1.22 (1.11 to 1.35)**  1.21 (1.11 to 1.33)** | REF  1.02 (0.90 to 1.17)  1.13 (0.96 to 1.33)  1.27 (1.02 to 1.57)  1.35 (1.10 to 1.66)**  0.98 (0.84 to 1.15)  1.25 (1.13 to 1.39)**  1.06 (0.94 to 1.19)  1.49 (1.23 to 1.80)**  1.12 (0.98 to 1.28)  1.09 (0.90 to 1.32) | REF  0.90 (0.78 to 1.04)  0.98 (0.82 to 1.17)  1.05 (0.74 to 1.49)  1.01 (0.87 to 1.19)  1.45 (1.14 to 1.84)**  1.51 (1.39 to 1.64)**  0.89 (0.77 to 1.02)  0.99 (0.72 to 1.37)  1.16 (0.98 to 1.37)  1.04 (0.87 to 1.23) |
| *State of residence and greenspace quartile interaction term*  1#California  2#Connecticut  2#Georgia  2#Iowa  2#Kentucky  2#Louisiana  2#Michigan  2#New Jersey  2#New Mexico  2#Utah  2#Washington  3#Connecticut  3#Georgia  3#Iowa  3#Kentucky  3#Louisiana  3#Michigan  3#New Jersey  3#New Mexico  3#Utah  3#Washington  4#Connecticut  4#Georgia  4#Iowa  4#Kentucky  4#Louisiana  4#Michigan  4#New Jersey  4#New Mexico  4#Utah  4#Washington | REF  0.98 (0.82 to 1.18)  1.06 (0.96 to 1.17)  0.88 (0.75 to 1.03)  0.89 (0.79 to 1.00)  1.11 (1.00 to 1.23)  0.99 (0.94 to 1.05)  1.07 (1.00 to 1.15)  0.98 (0.79 to 1.21)  1.18 (1.02 to 1.38)**  1.01 (0.94 to 1.10)  0.96 (0.87 to 1.06)  1.06 (0.96 to 1.18)  0.98 (0.82 to 1.17)  0.92 (0.80 to 1.06)  1.13 (1.01 to 1.25)**  0.92 (0.86 to 0.98)**  1.00 (0.92 to 1.08)  1.11 (0.94 to 1.33)  1.02 (0.85 to 1.22)  0.88 (0.80 to 0.97)**  0.98 (0.84 to 1.13)  1.13 (1.02 to 1.25)**  0.94 (0.77 to 1.15)  1.04 (0.89 to 1.22)  1.14 (0.98 to 1.32)  0.85 (0.79 to 0.92)**  0.98 (0.90 to 1.06)  0.91 (0.71 to 1.17)  1.11 (0.99 to 1.25)  1.01 (0.87 to 1.18) | REF  0.91 (0.78 to 1.06)  1.09 (0.90 to 1.30)  0.90 (0.66 to 1.23)  0.91 (0.64 to 1.29)  1.13 (0.89 to 1.45)  1.23 (1.09 to 1.40)**  1.03 (0.90 to 1.18)  0.86 (0.64 to 1.16)  1.22 (0.96 to 1.55)  1.15 (0.97 to 1.35)  0.87 (0.78 to 0.97)**  1.14 (0.95 to 1.37)  0.95 (0.67 to 1.35)  1.17 (0.90 to 1.54)  1.38 (1.16 to 1.65)**  0.95 (0.88 to 1.04)  1.00 (0.90 to 1.10)  0.99 (0.74 to 1.33)  1.34 (1.02 to 1.77)**  0.86 (0.72 to 1.04)  0.96 (0.77 to 1.21)  1.23 (1.01 to 1.49)**  0.90 (0.65 to 1.25)  1.37 (1.07 to 1.75)**  1.47 (1.13 to 1.91)**  0.88 (0.78 to 1.00)  0.99 (0.87 to 1.13)  0.87 (0.62 to 1.21)  1.15 (0.84 to 1.57)  1.23 (0.89 to 1.70) | REF  0.97 (0.73 to 1.29)  1.18 (0.93 to 1.48)  0.88 (0.59 to 1.32)  0.87 (0.68 to 1.13)  0.87 (0.66 to 1.13)  0.81 (0.71 to 0.92)  1.13 (0.91 to 1.40)  1.02 (0.66 to 1.57)  1.18 (0.82 to 1.71)  1.09 (0.92 to 1.28)  1.14 (0.93 to 1.41)  1.24 (1.02 to 1.51)**  1.13 (0.75 to 1.70)  0.85 (0.64 to 1.13)  0.84 (0.62 to 1.14)  0.98 (0.87 to 1.11)  1.06 (0.93 to 1.21)  0.95 (0.71 to 1.28)  0.98 (0.70 to 1.37)  1.14 (0.99 to 1.31)  1.20 (0.88 to 1.63)  1.53 (1.18 to 1.99)**  1.26 (0.67 to 2.36)  1.29 (0.99 to 1.69)  1.06 (0.75 to 1.49)  0.92 (0.78 to 1.08)  1.17 (0.92 to 1.49)  1.14 (0.59 to 2.22)  1.37 (0.96 to 1.95)  1.27 (0.85 to 1.88) |
| *Dual eligibility to Medicare and Medicaid*  Not eligible  Eligible (Poorer) | REF  1.41 (1.33 to 1.50)** | REF  1.36 (1.27 to 1.45)** | REF  1.52 (1.37 to 1.68)** |
| *Neighborhood SVI*  Low vulnerability  High vulnerability | REF  1.09 (1.03 to 1.16)** | REF  1.16 (1.08 to 1.25)** | REF  0.99 (0.88 to 1.12) |
| *Race and Ethnicity*  NHW  NHB  NHO  Hispanic | REF  0.96 (0.92 to 1.00)  0.60 (0.55 to 0.66)**  0.78 (0.73 to 0.84)** | REF  1.05 (0.98 to 1.14)  0.67 (0.59 to 0.76)**  0.86 (0.79 to 0.93)** | REF  0.95 (0.87 to 1.04)  0.50 (0.41 to 0.60)**  0.67 (0.57 to 0.77)** |
| *Comorbidity*  None  One  Two or more | REF  1.36 (1.31 to 1.41)**  2.23 (2.14 to 2.32)** | REF  1.06 (0.99 to 1.14)  1.35 (1.29 to 1.42)** | REF  1.80 (1.64 to 1.97)**  3.63 (3.25 to 4.04)** |

*BC: Breast cancer; CSHR=Cause specific hazard ratio; CVD: Cardiovascular disease; ACM: All-cause mortality; BCSM: Breast cancer specific mortality; CVDSM: Cardiovascular disease specific mortality; SVI: Neighborhood social vulnerability*

*All models are adjusted for race/ethnicity, SVI, dual enrollment (Medicare and Medicaid) eligibility, tract-level population density, comorbidity, and stratified by* *diagnosis year, stage, age, and tumor subtype. All models are adjusted for random error by census tracts clusters.*

*** : Denotes statistical significance (p≤0.05)*
